# Cross talk of vasopressin conditioned cell therapy in ischemic heart disease: Role of oxidative stress markers 

**DOI:** 10.22038/IJBMS.2022.62540.13837

**Published:** 2022-09

**Authors:** Mona Bagheri, Shakiba Nasiri Boroujeni, Hassan Ahmadvand, Afshin Nazari, Farzaneh Chehelcheraghi

**Affiliations:** 1 Student Research Committee, School of Medicine, Lorestan University of Medical Sciences, Khorramabad, Iran; 2 Medicinal Plants and Natural Products Research Center, Hamadan University of Medical Sciences, Hamadan, Iran; 3 Razi Herbal Medicines Research Center, Department of Physiology, Lorestan University of Medical Science, Khorramabad, Iran; 4 Cardiovascular Research Center, Shahid Rahimi Hospital, Lorestan University of Medical Sciences, Khorramabad, Iran; 5 Department of Anatomical Sciences, School of Medicine Lorestan University of Medical Sciences, Khorramabad, Iran

**Keywords:** Anti-oxidants, Catalase, Coronary vessels, Glutathione, Malondialdehyde, Mesenchymal stem cells, Myocardial Infarction, Myocardial ischemia, Oxidative stress, Vasopressins

## Abstract

**Objective(s):**

Background: Impaired coronary blood flow causes cardiac ischemia. Cellular therapy is a new approach to the treatment of myocardial ischemia. This study aimed to investigate the effect of adipose tissue-derived mesenchymal stem cells (AD-MSCs) conditioned with vasopressin on oxidative stress, perivascular collagen, and angiogenesis caused by myocardial infarction (MI) in rats.

**Materials and Methods:**

We divided 40 male albino Wistar rats into 4 groups; Control group; No intervention; in experimental groups, after it generated induced MI on models, it divided into three groups: Vehicle group (150 μl of cell-free culture medium received); ASC-MI group (6× 10^6^ AD-MSC received) and AVP-ASC-MI group (received 6 × 10^6^ AD-MSC conditioned with 10 nM vasopressin). Then, histologic parameters and anti-oxidant enzymes were evaluated 7 days post-MI cell injection.

**Results:**

Arterial muscle diameter improved and collagen deposition around the coronary arteries decreased in cell-received groups compared with the vehicle group. Malondialdehyde (MDA), catalase (CAT), (GSH) Glutathione, and Total Anti-oxidant Capacity (TAC) parameters were not significantly different between the cells received groups compared with the vehicle group. But the Catalase (CAT) parameter in the ASC-MI group had a significant increase from the control group.

**Conclusion:**

We prepared direct evidence that intramyocardial injection of AD-MSCs reveals the positive cardiac remodeling post-MI in rats, and these useful effects can be more enhanced by administrating injection of conditioned ADSCs with vasopressin.

## Introduction

Worldwide, more people die of cardiovascular disease (CVD) than any other cause, with approximately 40% of these deaths being related to coronary artery disease (CAD). It is the keystone of cardiac ischemia (a disorder of blood flow in the coronary arteries) (1). Cell therapy is a new approach in the treatment of ischemic heart disease because it has the potential to stimulate the regeneration of damaged heart cells. Also, stem cell therapy is used to restore the improvement of infarct-related arteries (2). Stem cells have great promise for tissue repair Regenerative medicine, and endothelial progenitor cells (EPCs) play an essential role in ischemic neovascularization. Adipose-derived mesenchymal stem cells (AD-MSCs) could be one of the cellular sources for medical applications (2, 3). Several studies, considered AD-MSCs *in vitro* because of their potential for direct differentiation into human heart cells and applied to the ischemic heart. 

During ischemia, the secretion of Matrix Metalloproteinases (MMPs) disrupts, causing collagen to accumulate around the coronary arteries (4, 5). AD-MSCs secret abundant anti-fibrotic factors and reduce the expression levels of collagen I, collagen III, and fibronectin, and prevent undesirable regeneration (6-9). But the limiting issue of cell therapy is their apoptosis because of stressful conditions, such as hypoxia. 

Arginine Vasopressin (AVP) or Antidiuretic Hormone (ADH) is a potent vasoconstrictor. It has positive effects on the heart and arteries. The effect of vasopressin causes contraction and expansion of coronary arteries, and, besides, the effect on coronary blood flow has mitogenic and metabolic effects on the heart. Regulation of vasopressin secretion and action thus represents a key homeostatic process that protects the osmotic milieu of the body, allowing normal cellular function such as effect on stem cells under hypoxic conditions (4). 

 Reactive oxygen species (ROS), by regulating vascular cell function, can play a central role in normal vascular physiology and significantly contribute to the development of the cardiovascular system. ROS plays a major role in the onset and progression of cardiovascular dysfunction associated with diseases, such as hyperlipidemia, diabetes mellitus, and hypertension (10). We believe that in ischemia, because of the reduction of mitochondrial membrane potential and disruption of the mitochondrial electron transport chain, an increase in electron leakage, and finally increased ROS production occurs (11). Overproduction of ROS is a fundamental mechanism of pathogenesis of endothelial dysfunction and cardiovascular disease, such as myocardial infarction (MI). Under MI, migration of inflammatory cells occurred, and vascular smooth muscle cells and endothelial cells produce ROS. 

Oxidative stress during ischemia leads to lipid peroxidation. To overcome the problem of ROS instability in measurement, malonaldehyde (MDA), a stable end product of lipid peroxidation, is often used as a marker of ROS production. Anti-oxidant scavenging systems defend organisms against ROS and inhibit oxidative damage [3]. The catalase (CAT), glutathione peroxidase (GSH), and total anti-oxidant capacity (TAC) of plasma are used as a scale of an organism’s ability to defend against ROS. 

Previous studies have found that, after MI, there is an increase in plasma MDA levels and a decrease in various anti-oxidants such as a-tocopherol, b-carotene, and vitamin C, suspend oxide dismutase and glutathione peroxidase (12, 13). The most effective therapy for the improvement of infarct-related arteries is primary coronary intervention (14, 15). AD-MSCs with their anti-oxidant effects show resistance to producing oxidative stress (16). After cell transplantation, AD-MSCs increase the activity of anti-oxidant enzymes. Thus, vasopressin-conditioned AD-MSCs may act as therapeutic tools in the face of oxidative stress (17). In the current study, we examined the serum levels of MDA production, CAT, GSH, and TAC, in rats with MI undergoing transplanted ASCs conditioning. We aimed to evaluate serum levels of oxidative stress to determine the relationship between oxidative stress parameters and the extent of myocardial tissue damage after treatment with conditioned and unconditioned stem cells. 

## Materials and Methods

***Animal model of MI***

The experimental procedures followed the organizational guidelines for the care and use of laboratory animals and approved by Lorestan University of Medical Sciences (ethical code IR. LUMS. REC.1400.056). 40 male albino Wistar rats weighing 250 to 300 g were divided into 4 groups. Animals in the control group had no intervention (intact heart), and in experimental groups after myocardial infarction (MI), recipients of culture medium (150 ml) as vehicle group, rat recipients of Adipose stem cells (6 × 10^6^) as ASC group and animals’ recipient of the conditioned Adipose stem cells with vasopressin (6 × 10^6^) as AVP-ASC group. All animals were given a rat chow diet, and water ad libitum and were housed under an alternating 12-hr/12-hr light/dark cycle.

 Experimental myocardial infarction was produced by ligation of the descending left coronary artery (LAD) (18, 19). A lateral thoracotomy was performed under anesthesia and the left coronary artery was looped by a single suture (5/0 nylon) at approximately 1 mm from its origin and gently tied. This procedure produced a demarcated area (cyanotic and bulging) of acute ischemia corresponding to the distribution of the left coronary artery distal to the occlusion. The chest was closed; rats were individually caged during a 24-hr recovery period (20).

***Equipment***

Echocardiography evaluated cardiac function noninvasively (software: ML750 Power Lab/4sp AD Instruments GE-Vingmed Ultrasound, USA); This ultrasound system is equipped with a 7-14 MHz probe.

***Two-dimensional echocardiography***

 Echocardiography was performed on animals who underwent mild anesthesia with ketamine at 10% (50 mg/kg) and Xylazine at 2% (10 mg/kg). Echocardiographic parameters were based on the main axis of the heart and were considered heart failure with an ejection fraction less than 50%. Cardiac output was estimated as (end-diastolic volume–end-systolic volume) × heart rate (ml/min) (Supplementary file Table1) (21).

***Cell culture and conditioned***

Human adipose-derived stem cells were purchased from the Iranian Biological Resource Center (IBRC) (code number: C0889). ASCs were isolated, characterized, and maintained in culture as previously described. Under this experimental condition, 28 μl of vasopressin was dispensed in a 7 ml culture medium containing DMEM (Gibco; 11885084) that includes low glucose concentrations and formulations without L-glutamine, 10% FBS, 1% Pen-Strep, and diluted 100 times. In brief, Cells were recovered and plated onto 10 cm culture plates. At 24-hour intervals, cultures were washed with PBS to take contaminating other unattached cells and refed with fresh medium. This process was repeated for three days (22, 23). Experimental myocardial infarction was performed as previously described. The left anterior descending (LAD) coronary artery was occluded, then 150 μl of serum containing 6 × 10^6^ AD-MSCs labeled with a fluorescent molecule (CM-DiI, C7000; Invitrogen) according to the manufacturer’s protocol was injected into the ischemic area (24).

***Serum collection and histological sectioning***

Animals were euthanized 7 days after cell transplantation, and the heart and sample of blood (approximately 3.5 ml) were collected and weighed. The serum was isolated by centrifuging at 4 °C, 15 min, 3000 rpm, and kept at -20 °C for biochemical evaluations. At the end of the procedures, hearts were rapidly removed and fixed in 10% formalin for 24 hr, embedded in paraffin, and histologically sectioned (5 mm). Samples were mounted onto slides and stained with hematoxylin, eosin, and Mason trichrome for measurement of the following histologic parameters at 400×magnification: 1. Area Occupied by Artery (AOA), 2. Muscle layer thickness in the arteries, 3. Number of capillaries in the six randomly selected fields (mm^2^), 4. Examining fibrosis, in per arterial tissue by collagen deposition (blue). All procedures were performed in samples obtained from middle segments of the ischemic heart border zone. Results were calculated of muscle layer diameter and cardiac fibrosis using the following formula and NIH ImageJ, version 1.42q software (NIH, Bethesda, MD, USA, based on the color histogram) (25, 26).

***Biochemical analysis***

*Evaluation of oxidative stress and inflammatory biomarkers*

The concentration of malondialdehyde (MDA), as the marker of lipid peroxidation, in the rat’s serum was measured based on the thiobarbituric acid (TBA) assay (27, 28), which was fully described in our previous study (29). The serum levels of GSH were determined spectrophotometrically at 412 nm according to Ellman’s method (30,31). Sinha’s method assayed the serum activities of catalase (CAT) (32). Glutathione peroxidase (GPX) activities were evaluated according to Rotruck *et al*.’s method (33). The measurements of blood serum TAC were performed using two spectrophotometric methods: the FRAS method (the ferric reducing ability of serum) originally described by Benzie and Strain (34) with some modifications (35) and the DPPH method (2.2-diphenyl-1-picryl-hydroxyl) (35). Results were calculated as a mean from three separate measurements (36-38).

*Statistical methods*

The SPSS statistical software (version 18; SPSS Inc., Chicago, Illinois, USA) was used for the analysis of data. Distributions of data in each experimental group were determined using the Kolmogorov-Smirnov test. Normal distribution data were compared using one-way ANOVA, followed by a *post hoc* LSD test, and nonnormal distribution by the Mann–Whitney U test. Results were presented as mean ± standard error (SE) and a *P*-value lower than 0.05 was accepted as statistically significant. All graphs were structured using GraphPad Prism version 8.0.2. (GraphPad Software Inc., USA).

## Results

***Human AD-MSC cardiac preservation and bio-distribution***

**Figure 1:**
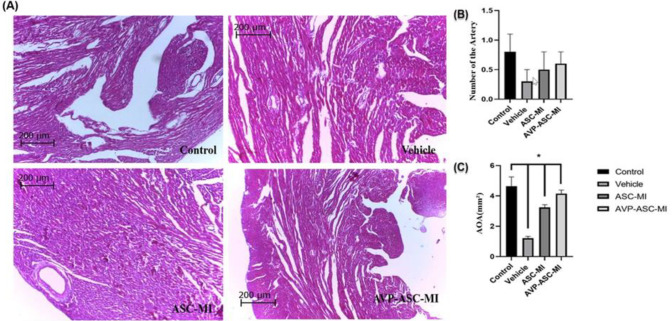
Conditioned treatment provided a relatively low inflammatory reaction and relatively high level of number and density of vessels micro-environment in infarcted hearts for transplanted MSCs to survive. (A) Representative HE staining images at the border zone 7 days after MI. Scale bar = 200 μm. (B) and (C) All data are mean ± SEM. Tukey’s test was performed with a one-way ANOVA followed by statistical analysis. *P<0.05, **P<0.01, ***P<0.001, and ****P<0.0001

**Figure 2:**
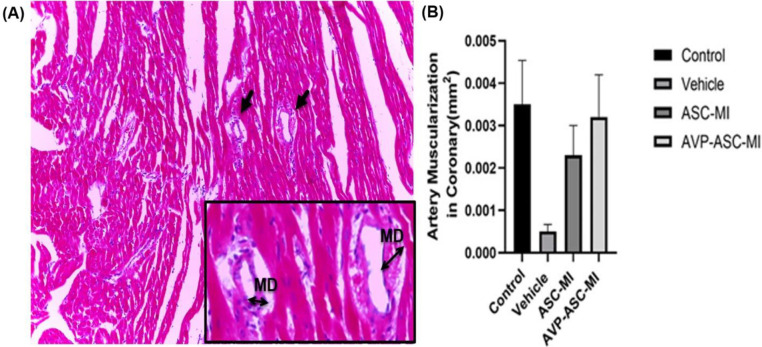
Muscular Diameter (MD) parameter of muscularization in coronary artery, Scale bar = 200, 50 μm. All data are mean ± SEM. Tukey's test was performed with a one-way ANOVA followed by statistical analysis. *P<0.05, **P<0.01, ***P<0.001, and ****P<0.0001

**Figure 3:**
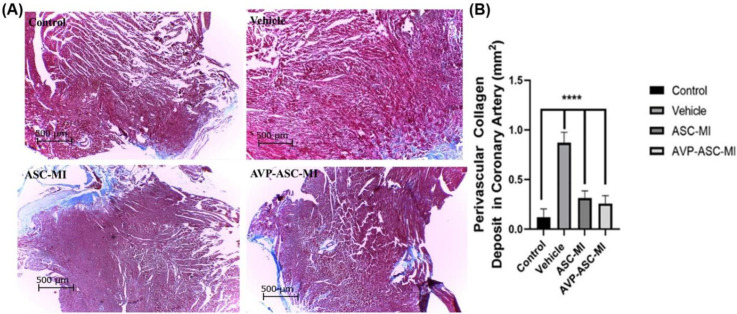
Conditioned and MSCs synergistically improved cardiac function and improved fibrosis after MI. (A) Representative transverse heart sections analyze with Masson trichrome staining at 7 days after MI. Scale bar = 10 µm. Blue, myocardium; image j software performed shiny blue, scarred fibrosis of the perivascular in coronary arteries, (B) Quantification of Staining images for collagen analysis in each group. All data are mean ± SEM. We performed statistical analysis with two-way ANOVA, followed by the Tukey *post hoc *test. *P<0.05, **P<0.01, ***P<0.001, and ****P<0.0001

 On day seven, the fluorescent-labeled AD-MSCs were found in the infarct border zone of myocardial infarction in ASC-MI and AVP-ASC-MI groups by Labomed 400×magnification EPI-Fluorescence Microscope. Evaluation of arterial density at a probe (12×12cm), using imaging of hematoxylin-eosin-stained tissue sections under a light microscope, 40×magnification was done. The Mean±SEM of Arterial density in the vehicle and control group; the lowest and the highest, respectively 0.4±0.516 and 1.1+0.56. Evaluation of Area Occupied by Artery (AOA) (mm²) revealed Mean±SEM of the vehicle and control groups; the lowest and the highest were respectively 0.02593±0.0117 and 0.05199±0.0282 (Figure 1). The means muscularization Arterial (MA) level was calculated in each of the experimental and control groups. The lowest and the highest Mean ± SEM, respectively were related to vehicle (0.0016±0.00237) and control (0.0047±0.00236) groups (Figure 2). To evaluate the amount of collagen deposition around the coronary arteries (CD), tissue sections stained with Mason trichrome (mm²) were used and photographed with light microscopy 40×magnification. The Mean±SEM of collagen deposition in the control and vehicle groups was the lowest and the highest (respectively; 0.00192±0.0025 and 0.00591±0.0059). Other groups were not significantly different (Figure 3).

***Biochemical analysis*** 

Concentrations of oxidative stress metabolites in blood serum were identified. The mean ±SEM of MDA concentration in the vehicle group was 0.308±0.0173, which was the highest level between groups. The standard deviation values between the groups were 0.0025 to 0.0032, showing that the groups were not significantly different, *P*=0.6891. Mean±SEM of serum GSH concentration in the control group was 2.748±0.741, the lowest and the highest were related to the AVP-ASC-MI group at 3.687±0.739, *P*=0.6970. The standard deviation of the groups was between 0.23 and 0.267 and they did not differ significantly from each other. Mean ±SEM serum activities of CAT, in AVP-ASC-MI (lowest) and ASC-MI (highest) were 200.354±82.579 and 387.797±95.453, respectively, *P*=0.9184. The standard deviation values were between 26.1 and 39.52, which were approximately equal. The mean ±SEM of the TAC value control group was 1.4968±0.059, the lowest, and the ASC-MI group was 1.5455±0.044, the highest, *P*=0.6930. The standard deviation of the groups was between 0.0142 and 0.0195, showing that the groups were not significantly different. In the study of blood serum of the studied animals, the Mean ±SEM of the concentration of oxidative stress metabolite MDA was the highest vehicle average, 0.308±0.0173. The standard deviation values between the groups were 0.0025 to 0.0032, showing that the groups were not different, *P*=0.6891. Mean±SEM of serum GSH concentration in the control group with 2.748±0.741 was the lowest and the AVP-ASC-MI group at 3.687±0.739 was the highest, (*P*=0.6970). The standard deviation of the groups was between 0.23 and 0.267 and they did not differ significantly from each other. Remarkably Mean ±SEM serum activities of CAT, in AVP-ASC-MI (lowest) and ASC-MI (highest) are 200.354±82.579 and 387.797±95.453, respectively (*P*=0.9184). The standard deviation values are between 26.1 and 39.52, which are approximately equal. Also, the study of confidence intervals shows that the ASC-MI group has a significant difference from all groups. Also, the Mean ±SEM of the TAC value control group is 1.4968±0.059 (lowest) and that of the ASC-MI group is 1.5455±0.044 is the highest, *P*=0.6930. The standard deviation of the groups was numerically between 0.0142 and 0.0195 and showed that the groups were not significantly different.

**Table 1:** Mean ±SEM of biochemical parameters for the effects of Vasopressin conditioned cell therapy on the serum levels of MDA, GSH, CAT, and TCA in ischemic heart disease in rats

Parameters	Control	Vehicle	ASC-MI	AVP-ASC-MI	*P*-Value
**MDA**	**0.3±0.007**	**0.3084± 0.017**	**0.295± 0.008**	**0.307±0.010**	***P*** **=0.** **6891**
GSH CAT TCA	2.748±0.741 0.30±.00789 1.496± 0.059	3.377±0.846 0.3084± 0.017 1.5225± 0.052	3.45±0.745 0.295± 0.008 1.545±0.044	3.687±0.739 0.307±0.010 1.486± 0.0617	*P*=0.6970 *P*=0.9184 *P*=0.6930

Malondialdehyde (MDA; mmol Trolox equivalent/lit)), Catalase (CAT; Unit/mg protein), Glutathione (GSH; Micromole/mg-protein), and Total Anti-oxidant Capacity (TCA; mmol Trolox equivalent/lit) parameters

## Discussion

Recent studies have shown that cardiovascular risk factors, such as MI, correlate with the number and function of arterial vessels. Understanding the mechanisms that regulate endothelial cells’ function may provide new insights into the pathogenesis of vasculogenesis and may lead to the development of specific treatments to prevent ROS production and ultimately correct vascular dysfunction. 

We have shown that arterial histological parameters improve vascular function through anti-oxidant mechanisms. In the present study, we describe our current understanding of the contribution of oxidative stress to stem cells with vasopressin-conditioned medium and vascular dysfunction in cardiovascular disease (39, 40). Our focus is on the potential mechanisms that underlie oxidative stress-induced damage and stem cells.

Early renovation of blood flow to the myocardium infarction is the only way to prevent progression to myocardial necrosis and thus limit the infarct size. However, a sudden renovation of oxygen supply to previously ischemic myocardium can lead to oxidative stress, with consequent oxidative injury to cells’ function and structure because of lipid peroxidation. Several studies have evaluated the level of MDA in patients with MI after treatment (41). In all cases, there was an increase in MDA after therapy, although, in some studies, the increase did not reach the level of statistical significance. Also, there were differences in the time course of changes in the MDA level. We examined the status of oxidants and anti-oxidants with changes in CAT, GSH, TAC, and MDA levels following MI and conditioned stem cell transplantation.

 MDA levels in the vehicle group were higher than in the control group, and TAC levels were lower. This shows that oxidative stress is elevated during ischemia, which agrees with previous studies. We did not find any correlation between oxidative stress parameters (MDA and TAC) and serum enzyme activity, showing that these aspects of oxidative stress do not depend on the extent of damage to the myocardium (42).

Similarly, Berg *et al*. did not find a direct relationship between 8-iso-PGF2a, a marker of oxidative stress, and troponin T, a marker of myocardial injury. The duration of coronary occlusion, evaluated as the time from the occurrence of the symptoms of MI to the opening of an infarct-related artery, also did not correlate with the serum MDA and CAT, GSH, and TAC levels. 

We reported an increase in MDA levels during the prolongation of ischemia in rat hearts; in our research, the duration of ischemia was evaluated by the history of pale. It is unknown whether prolonged ischemia (i.e., lasting a few hours) leads to a continuous rise in MDA levels or whether the level reaches a plateau. All previous studies reported an increase in the MDA levels following the opening of infarct-related arteries (15). Although we expected a rise in the plasma MDA level, it fell significantly after stem cell therapy. Olsson *et al*. reported similar findings. 

We found an immediate and significant reduction in TAC after MI, but in ASC-MI it was higher than in control and other experimental groups. The decrease in TAC after MI may show a depletion of anti-oxidants because of the overproduction of ROS in the damaged area.

A decrease in TAC after cell therapy suggests increased ROS production, but the decrease in MDA failed to document increased lipid peroxidation in our animals. It is unlikely that additional medical treatments influenced our results. 

We showed the efficiency of the decrease in hydrogen peroxide levels in cases of oxidative stress in the cell cultures. Overexpression of CAT protected endothelium of the human aorta against apoptosis caused by the oxidized forms of low-density lipoproteins (ox-LDL), in our research CAT increased in the ASC-MI group, reversely in AVP-ASC-MI group it decreased. In addition, in both groups, the GSH levels were increased. In the future, these results may provide the key treatment for human heart failure; they confirm the efficacy of the combined effect of SOD and CAT activity in blocking oxidative stress. Meanwhile, the conjugation of SOD or CAT with antibody to platelet-endothelial cell adhesion molecule-1 (PECAM-1) provided a versatile molecular tool for testing the role of reactive oxygen species in vascular pathology. Anti-PECAM/SOD, but not anti-PECAM/CAT, inhibited vascular endothelial growth factor (VEGF)-induced increase in endothelial permeability. 

This has identified a crucial role for endogenous superoxide radicals in VEGF-mediated regulation of endothelial barrier function. Anti-PECAM/CAT, but not anti-PECAM/SOD, alleviated endothelial hyper-permeability, implicating primarily hydrogen peroxide in the disruption of the endothelial barrier. Targeting the anti-oxidant enzymes to endothelial cells offers a future perspective for the development of effective cardioprotective remedies (43). Our analysis showed an independent association between GSH and the risk of cardiovascular events, after adjusting for potential confounding variables. 

The analysis of SOD activity provided further confirmation of the role of impaired anti-oxidant status in facilitating cardiovascular disease, although catalase showed no predictive value of cardiovascular events. Among the 3 anti-oxidants, GSH showed the best predictive value, as it was the only enzyme significantly associated with cardiovascular events in the fully adjusted model. For this purpose, we measured the activity of three anti-oxidant enzymes, namely glutathione (GSH) and catalase, which catabolize hydrogen peroxide 8 and superoxide dismutase (SOD), which converts superoxide anion to hydrogen peroxide 9.

 In our research, conditioning for transplanted cells led to increased cell survival in the tissue after transplantation. Many studies are performed on conditioning stem cells with vasopressin under hypoxic conditions. Vasopressin is a nano-peptide that is enhanced in congestive heart failure and has a protective role in heart damage. In cardiac ischemia, there is a decrease in arterial density because of hypoxia and lack of expression of angiogenesis factors. But AD-MSCs increase arterial density by having the potential to secrete angiogenesis factors and express VEGF (43). Hypoxia was a potent stimulus for the angiogenic activity of AD-MSCs (44). 

We showed treatment with ASCs and AVP-ASCs transplanted cells in the ischemic region would influence angiogenesis and increase the muscular diameter of the arteries. Lee *et al*. showed that the expression of the α-SMC Actin marker is present in AD-MSCs and causes the formation and increase of smooth muscle in the arteries (45). Also, recent research revealed that treatment with ASCs and AVP-ASCs would reduce anti-fibrotic factors and finally decrease perivascular collagen deposition. Ischemic conditions can overshadow the endogenous anti-oxidant system and cause more tissue damage. Treatment with ASCs and AVP-ASCs in cardiac ischemia would increase the function of anti-oxidant enzymes and prevent overexpression of oxidative stress metabolites.

## Conclusion

The findings of this study showed treatment with AVP-ASCs has a relatively greater effect on tissue factors in the experimental models of MI. ASCs cause growing muscle diameter and reduced collagen deposition in coronary arteries. This approach may represent a promising alternative strategy for cardiovascular disease treatment. Change in the activity of anti-oxidant enzymes in the bloodstream of experimental groups provides evidence that supports the role of the anti-oxidant system in the activation of vasopressin against oxidative stress. This result could be a defense mechanism in pathological conditions derived from myocardial infarction.

## Authors’ Contributions

FCH Supervised, conceptualized, provided methodology, analyzed or interpreted the data, and wrote, reviewed, and edited the manuscript. AN Supervised the design of the heart attack model. HA Contributed to the biochemical studies. MB Participated in the histological and biochemical studies and contributed to the statistical analysis. SN Contributed to cardiac surgery and cell differentiation technique. All authors read and approved the final manuscript.  

## Conflicts of Interest

The authors declare that they have no competing interests.
